# The Medical Annual

**Published:** 1887-06

**Authors:** 


### THREE YEAR-BOOKS.

*The Medical Annual.* 1887. Pp. 550. Bristol: J. WRIGHT AND CO.

*Cassell's Year-Book of Treatment.* 1886.

*Official Year-Book of the Scientific and Learned Societies.* Pp. 236. London: C. GRIFFIN & CO.

*The Medical Annual* for this year gains in value in one department by diminishing its value in another. The Therapeutic matter is greatly increased in extent, while its very useful summary of the Spas of Europe has been abolished. The former change places it in competition with *Cassell's Year-Book*, which is unnecessary, to say the least of it; while the latter change is a distinct loss.

*Cassell's Year-Book* retains its character of high excellence, as might be expected, from the position and reputation of the men engaged in its production. With a few of the collaborateurs its pages seem to be used rather for the purpose of criticism than for summarising and abstracting. This we consider ought to be strictly forbidden.

*The Official Year-Book of the Scientific and Learned Societies* seems to have crystallised into something like permanent and definite form. To the working scientific man it is a very valuable, almost necessary, addition to his stock of books. It is so full that we are sorry that it is not absolutely complete and exhaustive. We should like to find in future issues the days of meeting of the societies, and the names of the papers read at every one of them. Possibly this may overstep the limits of the publishers' means, and the patience of the secretaries of the societies.

